# Identification of an IGHV3-53-Encoded RBD-Targeting Cross-Neutralizing Antibody from an Early COVID-19 Convalescent

**DOI:** 10.3390/pathogens13040272

**Published:** 2024-03-23

**Authors:** Yuanyuan Hu, Caiqin Hu, Shuo Wang, Li Ren, Yanling Hao, Zheng Wang, Ying Liu, Junwei Su, Biao Zhu, Dan Li, Yiming Shao, Hao Liang

**Affiliations:** 1 Guangxi Key Laboratory of AIDS Prevention and Treatment & Biosafety III Laboratory, Guangxi Medical University, Nanning 530021, China; 2 National Key Laboratory of Intelligent Tracking and Forecasting for Infectious Diseases, National Center for AIDS/STD Control and Prevention, Chinese Center for Disease Control and Prevention, Beijing 102206, China; 3 State Key Laboratory for Diagnosis and Treatment of Infectious Diseases, National Clinical Research Center for Infectious Diseases, National Medical Center for Infectious Diseases, Collaborative Innovation Center for Diagnosis and Treatment of Infectious Diseases, The First Affiliated Hospital, Zhejiang University School of Medicine, Hangzhou 310003, China

**Keywords:** neutralizing antibody, SARS-CoV-2, Omicron subvariants, RBD

## Abstract

Since November 2021, Omicron has emerged as the dominant severe acute respiratory syndrome coronavirus 2 (SARS-CoV-2) variant, and its sublineages continue to appear one after another, significantly reducing the effectiveness of existing therapeutic neutralizing antibodies (NAbs). It is urgent to develop effective NAbs against circulating Omicron variants. Here, we isolated receptor binding domain (RBD)-specific single memory B cells via flow cytometry from a COVID-19 convalescent. The antibody variable region genes of the heavy chain (VHs) and light chain (VLs) were amplified and cloned into expression vectors. After antibody expression, ELISA screening and neutralizing activity detection, we obtained an IGHV3-53-encoded RBD-targeting cross-neutralizing antibody D6, whose VL originated from the IGKV1-9*01 germlines. D6 could potently neutralize circulating Omicron variants (BA.1, BA.2, BA.4/5 and BF.7), with IC50 values of less than 0.04 μg/mL, and the neutralizing ability against XBB was reduced but still effective. The KD values of D6 binding with RBD of the prototype and BA.1 were both less than 1.0 × 10^−12^ M. The protein structure of the D6-RBD model indicates that D6 interacts with the RBD external subdomain and belongs to the RBD-1 community. The sufficient contact and deep interaction of D6 HCDR3 and LCDR3 with RBD may be the crucial reason for its cross-neutralizing activity. The sorting and analysis of mAb D6 will provide important information for the development of anti-COVID-19 reagents.

## 1. Introduction

Severe acute respiratory syndrome coronavirus 2 (SARS-CoV-2) has resulted in a global pandemic [1], causing more than 774 million infections and more than 7 million fatalities since its initial outbreak in December 2019 (https://covid19.who.int/, accessed on 8 January 2024). Nabs have played an important role in the prevention and control of severe cases of SARS-CoV-2 infection [2,3]. Meanwhile, effective Nabs can guide the design of SARS-CoV-2 vaccine immunogens [4,5,6,7]. However, SARS-CoV-2 continues to mutate, leading to the emergence of a large number of variants that pose significant challenges to previous NAbs. 

During the initial process of SARS-CoV-2 infecting cells, SARS-CoV-2 binds to the host receptor angiotensin-converting enzyme 2 (ACE2) via the receptor-binding domain (RBD) of its spike (S) glycoprotein, tethering the viral particle to the cell surface [8,9]. This receptor binding process then triggers S-mediated membrane fusion, ultimately leading to the entry of the virus to establish an infection [10]. Therefore, RBD is the main target of host humoral immunity [11]. Multiple SARS-CoV-2 RBD-targeting NAbs have been developed to fight against viral infection, and several NAbs have been approved by the FDA (Food and Drug Administration) of the United States or EMA (European Medicines Agency) for urgent treatment of COVID-19 [12,13,14]. However, Omicron has become the major dominating and circulating SARS-CoV-2 variant worldwide nowadays [15]. Omicron has an unusually large number of mutations compared with the wild strain. More than 50 mutations were found in Omicron, with more than 30 mutations located on the S protein, of which about 15 mutations were located on the RBD [16,17,18], including G339D, S371L, S373P, S375F, K417N, N440K, G446S, S477N, T478K, E484A, Q493R, Q496S, Q498R, N501Y and Y505H, and among these, only K417N, T478K, and N501Y often appear in previous variants of concern (VOCs) [19]. Shortly thereafter, Omicron evolved into a number of different subvariants, such as BA.1, BA.2, BA.3, BA.4/5, BF.7, XBB, EG.5, etc. [20]. These subvariants are often more transmissive and more resistant to previously obtained NAbs [21]. They show significant resistance to most of these approved NAbs, including imdevimab, casirivimab, bamlanivimab, etesevimab, regdanvimab and sotrovimab [17,22,23,24,25]. It has also been reported that Omicron escaped from over 85% of the tested neutralizing antibodies [18]. Huang et al. reported that 37 out of 50 RBD-targeting mAbs lost their neutralizing activity toward Omicron, while others, such as BD-629, P2C-1F11, S2E12 and S309, retained their neutralizing activity, but displayed variably decreased activities against the Omicron subvariants [26]. These data indicate that there is an urgent need to isolate and identify new broadly neutralizing antibodies against circulating SARS-CoV-2 variants and future pandemic variants of SARS-CoV-2.

In this study, we isolated one cross-neutralizing antibody from an early COVID-19 convalescent by a single memory B cell sorting strategy. We analyzed the antibody gene characteristics and binding epitope, and tested its neutralizing activity against the prototype and several widespread Omicron subvariants. In addition, we tried to explain its cross-neutralizing mechanism by simulating and analyzing the antigen–antibody structure. 

## 2. Materials and Methods

### 2.1. Study Subject 

A COVID-19 convalescent (donor YYQ), who had recovered from SARS-CoV-2 infection at the first affiliated hospital, the Zhejiang University School of Medicine, was recruited in our study. Donor YYQ was confirmed positive to COVID-19 according to “Diagnosis and treatment of novel coronavirus pneumonia” in January 2020 and hospitalized for 10 days. Eighteen months after leaving hospital, donor YYQ received one dose of inactivated COVID-19 vaccine BBIBP-CorV. Five months later, 5 mL of whole blood was sampled (Table 1). This study was reviewed and approved by the Ethics Committee of the First Affiliated Hospital, Zhejiang University School of Medicine. Written informed consent was provided.

### 2.2. Construction of RBD Probe

SARS-CoV-2 RBD (GenBank No: MN908947) with Avi-tag sequence was constructed in pDRVI1.0 vector. After expression and purification, the RBD proteins were biotinylated using a biotin ligase Bir A500 kit (Avidity, San Diego, CA, USA) according to the manufacturer’s instruction. To conjugate RBD with streptavidin-phycoerythrin (SA-PE) (Sigma, St. Louis, MO, USA), 1/5 of the molar equivalent of the SA-PE was added to biotinylated RBD and incubated on ice for 20 min with gentle rocking. The preceding step was repeated 5 times until the molar ratio of SA-PE and RBD reached 1:1. Finally, the RBD with PE (RBD-PE) was stored at 4 °C, avoiding light.

### 2.3. Isolation of RBD-Specific Single B Cells by Flow Cytometry

Probe-specific single B cells were sorted as previously described [27]. Briefly, one tube of PBMCs was thawed in water at 37 °C and resuspended in a prewarmed RPMI-1640 (Corning, Corning, NY, USA) containing 10% fetal bovine serum (Sera Pro, Gremlingen, BW, Germany) and 50 IU/mL benzonase (Sigma, St. Louis, MO, USA). After washing with PBS, the PBMCs were stained with a LIVE/DEAD dye (Invitrogen, Carlsbad, CA, USA) on ice for 20 min. After washing, the PBMCs were stained with an antibody cocktail containing anti-CD3-Pacific Blue, anti-CD8-Pacific Blue, anti-CD19-BV510, anti-IgM-PercpCy5.5, anti-IgG-FITC, anti-CD27-APC-Cy7, anti-PD-1-PECy7, anti-CXCR5-APC-R700, anti-CXCR3-PECy5, anti-CD45RA-BV650, anti-CD4-BV605 (above antibodies are all from BD Biosciences), anti-CD14-eflur450 (ebioscience, USA), anti-CD20-ECD (Beckman Coulter, Bria, CA, USA) and RBD-PE. After filtering through a cell strainer (Corning, Corning, NY, USA), the PBMCs were sorted on BD FACS Aria SORP. RBD-specific single memory B cells were gated as CD3^−^, CD8^−^, DAPI^−^, CD14^−^, CD19^+^, CD20^+^, CD27^+^, IgM^−^, IgG^+^, RBD-PE^+^, and were sorted into a 96-well PCR plate containing cell lysis buffer. Then, the PCR plate was frozen immediately at −80 °C and stored overnight. 

### 2.4. MAb Gene Amplification, Vector Construction, and mAb Expression

After cell lysis and reverse transcription of RNA, the VHs and VLs were amplified by nest PCR as previously described [28]. Then the VHs and VLs were cloned into eukaryotic transient expression vectors containing corresponding constant regions of IgG1 heavy chains and light chains. Equal amounts of paired heavy and light chain plasmids were co-transfected into 293F cells with polyethyleneimine. After five days incubation at 37 °C with 5% CO_2_, antibodies were purified from the culture supernatant using protein A columns (Senhui Microsphere, Beijing, China). The concentration of antibody was determined using a Nanodrop 2000 ultramicro spectrophotometer (Thermofisher, Waltham, MA, USA). The size of the MAb protein was determined by denaturing sodium dodecyl sulfate polyacrylamide gel electrophoresis (SDS-PAGE).

The gene characteristics of VHs and VLs were analyzed on the IMGT/V-QUEST website (www.imgt.org/IMGT_vquest/vquest), including the germline gene, the somatic hypermutation rate (SHM) and the length of the CDR loop. Sequences of VHs and VLs were aligned by Bioedit 7.0.5.3 software respectively.

### 2.5. Enzyme-Linked Immunosorbent Assay (ELISA)

The ELISA experiment was conducted as previously reported [29]. The ELISA plate was coated with 100 μL of the RBD (1 µg/mL in PBS) at 4 °C overnight. The plate was washed with PBST (PBS containing 0.2% Tween 20) and blocked with 300 µL of blocking buffer (5% skim milk plus 2% bovine serum albumin in PBS) at room temperature for 2 h. The samples were 5-fold serially diluted, added to the plate after washing and incubated at 37 °C for 1 h. After the plate was washed, goat anti-human IgG labeled with HRP (Sino Biological, China) diluted 1:5000 with antibody diluent was added to the plate and incubated for 1 h at 37 °C. After washing, 100 μL of TMB substrate (Kinghawk, Anhui, AH, China) was added and the plate was incubated at 37 °C for 5 min. Finally, 50 μL of stop solution (2 M H_2_SO_4_) was added and the optical density (OD) was measured by a spectrophotometer at 450 nm. 

### 2.6. SARS-CoV-2 Pseudovirus Neutralization Assays

Neutralization activity was measured as described previously [30]. Briefly, mAb or plasma were serially diluted and incubated with pseudovirus in duplicate for 1 h at 37 °C. Cell control and virus control were set. Then, freshly trypsinized Huh-7 cells were added to each well. After 24 h of incubation at 37 °C with 5% CO_2_, 150 µL of the supernatant was removed from each well, and 100 µL of luciferase reagent (Promega, Madison, WI, USA) was added. Two minutes later, 150 μL of lysate was transferred to a black plate for the detection of luminescence using a Victor 3 luminometer (PerkinElmer, Waltham, MA USA). The 50% inhibitory dose or inhibitory concentration (ID50 or IC50) was calculated as the plasma dilution ratio or mAb concentration that caused a 50% reduction in relative luminescence units (RLU) compared with the level of the virus control subtracted from that of the control cell. 

### 2.7. Authentic SARS-CoV-2 Neutralization Assays

The authentic SARS-CoV-2 neutralization assay was described previously [31]. Briefly, the mAb, which was two-fold serially diluted, was incubated with the same volume of 100 TCID50 of SARS-CoV-2 at 37 °C. The mixtures were then transferred to a monolayer of Vero-E6 cells in a 96-well plate. After culturing at 37 °C for 4 days, cytopathic effects (CPE), which are caused by the virus infection, were calculated for each well. The percentage of neutralization and corresponding antibody concentration were plotted using nonlinear regression using GraphPad Prism 9. The IC50 of mAb was determined.

### 2.8. Biolayer Interferometry (BLI)

The binding kinetics of mAb to the RBD protein were measured with an Octet RED96 instrument as previously described [29]. In brief, 5 μg/mL of biotinylated RBD in kinetic buffer (0.02% Tween-20% and 0.1% Albumin bovine serum in PBS) was loaded onto the surface of the SA capture biosensor (ForteBio, Dallas, TX, USA). After washing with kinetic buffer, the biosensor was exposed to two-fold serial dilutions of mAb for 120 s to determine the association constant and then to a kinetic buffer for 300 s to obtain the dissociation constant. The association constant and dissociation constant were calculated by a global fitting method with Data Analysis HT 9.0 software.

### 2.9. Structure Analysis of Antibody-Antigen Complex

The protein structure models of the VH and VL of D6 were built via I-TASSER (https://zhanggroup.org/I-TASSER/) [32]. The crystal structure of SARS-CoV-2 RBD binding with CC12.1 (PDB: 6XC2) was downloaded from the Protein Data Bank (https://www.rcsb.org). After structure superimposition of D6 with the RBD-CC12.1 complex by the PyMOL2.4.1 software, we could analysis the characteristics of their structure and label the antibody segments and amino acid sites.

## 3. Results

### 3.1. The Plasma of Donor YYQ Not Only Contains RBD-Targeting Antibodies but Also Contains Neutralizing Antibodies against Multiple SARS-CoV-2 Variants

The blood sample was collected from a COVID-19 convalescent (donor YYQ), who had recovered from mild-type symptoms, and his basic information is listed in Table 1. The plasma of YYQ bound well to prototype RBD with the end-titer of 1:400 in the ELISA experiment, indicating that the plasma has RBD-targeting antibodies. In the neutralization experiments, the plasma of YYQ could neutralize SARS-CoV-2 prototype pseudovirus, with an ID50 value of 540, and could neutralize SARS-CoV-2 authentic viruses, including the prototype, Bata (B.1.351), Gamma (P.1) and Kappa (B.1.167.1), with ID50 values of 384, 12, 4 and 16, respectively (Table 2), suggesting that the plasma possesses neutralizing antibodies against SARS-CoV-2. 

### 3.2. RBD-Specific Memory B Cell Sorting and Antibody Cloning

**Figure 1:**
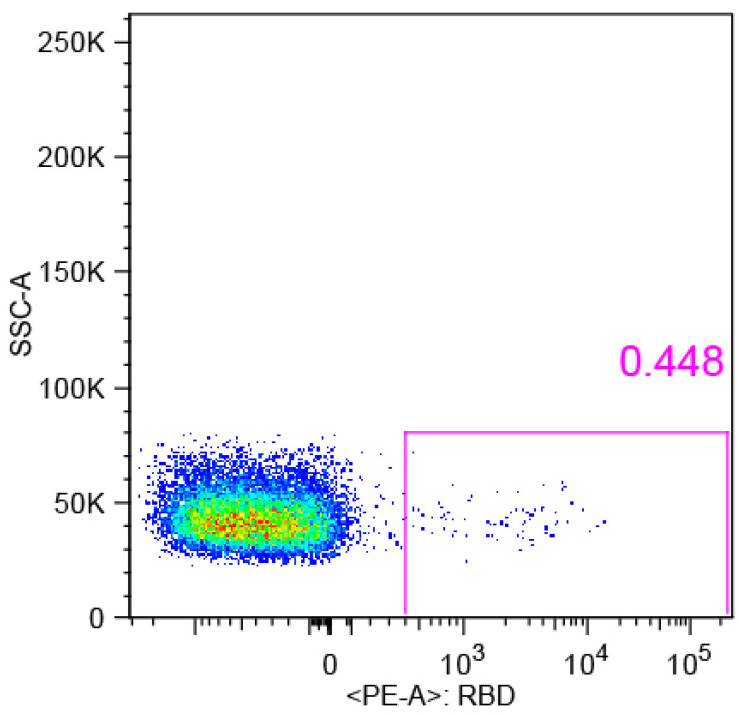
Flow cytometry graph. Single SARS-CoV-2 RBD-specific B cells were sorted using a FACS Aria SORP. One hundred and fourteen memory B cells which bound with RBD-PE were sorted into a 96-well plate containing lysis buffer. The colors represent cell concentration, and the cell concentration decreases from red, yellow, green, to blue.

To isolate the RBD-specific memory B cells, the PBMCs of donor YYQ were stained with an antibody panel and a probe (RBD-PE), and then sorted via BD FACS Aria SORP. In total, 114 antigen-positive memory B cells were sorted to rescue native heavy and light chain pairs for mAb production and functional verification (Figure 1).

**Figure 2:**
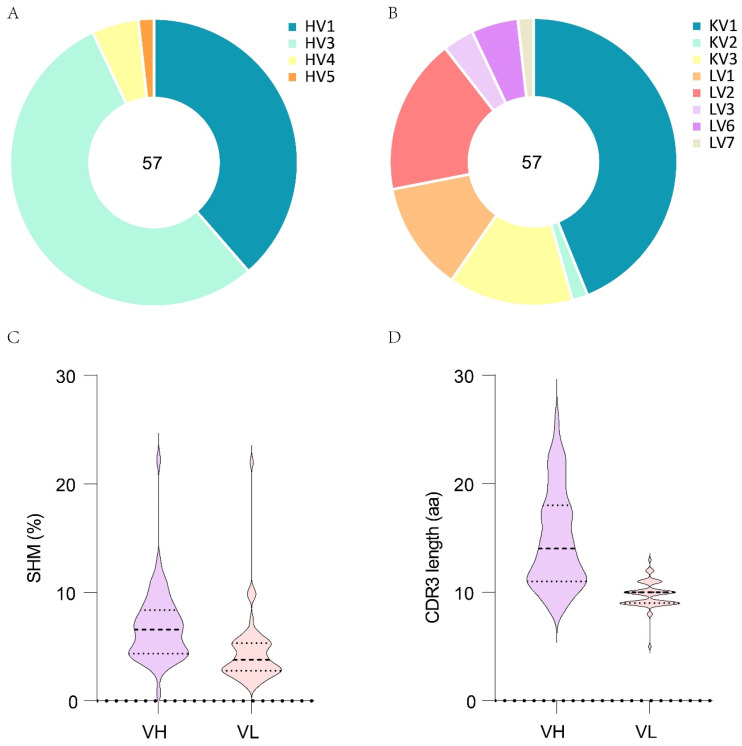
Sequence characteristics of antibodies isolated from donor YYQ. The gene family usages of VH (**A**) and VL (**B**) for the 57 antibodies. (**C**) Rates of nucleotide substitutions in VH and VL for the 57 antibodies. (**D**) The amino acid lengths of the CDR3 loop of VH and VL for the 57 antibodies.

After cell lysis, reverse transcription and gene amplification, 57 natural pairs of VH and VL genes were obtained. The sequence characteristics of VH and VL were analyzed by the IMGT/V-QUEST. These VH genes originated from IGHV1 (38.6%), IGHV3 (54.4%), IGHV4 (5.3%) and IGHV5 (1.8%) (Figure 2A), while the VL genes mainly originated from IGKV1 (43.9%), IGLV2 (17.5%), IGKV3 (14.0%) and IGLV1 (12.3%) (Figure 2B). The SHM and CDR3 length are important characteristics of mAb, which may represent the degree of mAb maturity. The average SHM rates of VH and VL were 6.82% and 4.45% (Figure 2C), while the average CDR3 lengths of VH and VL were 14.79 aa and 9.65 aa, respectively (Figure 2D).

After plasmid construction and protein expression, 57 mAbs were obtained. Firstly, we tested the binding activity of mAbs to prototype RBD and Omicron RBD, respectively. Thirty-nine mAbs potently bound to prototype RBD, and eleven mAbs bound to Omicron RBD (Supplementary Table S1). Then we tested the neutralizing activity of nine mAbs which could bind to both prototype RBD and Omicron RBD. Of these mAbs, only D6 could neutralize the prototype strain.

### 3.3. The Gene Characteristics of D6 

**Figure 3:**
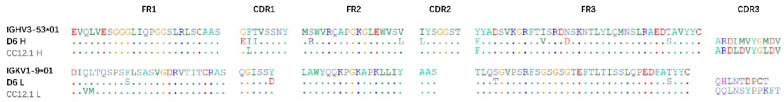
Amino acid sequence comparisons of the mAbs heavy chain and light chain with alignment to their respective germline chain. The framework region (FR) and complementarity determining region (CDR) were determined using the IMGT/V-QUEST program. The symbol ‘.’ denotes the same amino acid. Different colors represent different amino acids, which are automatically generated by Bioedit 7.0.5.3 software.

**Figure 4:**
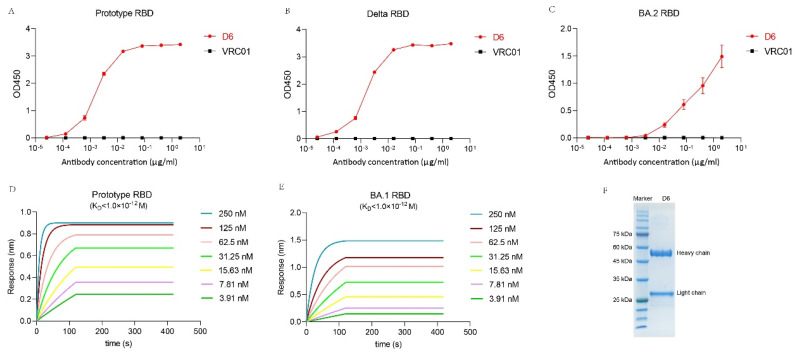
Binding activity and binding kinetics of D6 with SARS−CoV−2 RBD. Binding activity of D6 with RBD of the prototype (**A**), Delta (**B**) and BA.2 (**C**) were measured by ELISA experiments. Binding kinetics of D6 with RBD of the prototype (**D**) and BA.1 (**E**) were measured by BLI. Denatured mAb D6 was detected by PAGE electrophoresis (**F**).

The D6 heavy chain variable, diversity, and joining (IGHV, IGHD, and IGHJ) regions are encoded by the germline genes IGHV3-53*01, IGHD2-8*01, and IGHJ6*02, and the light chain variable and joining (IGKV and IGKJ) regions are encoded by IGKV1-9*01 and IGKJ3*01, respectively. It has been reported that IGHV3-53-encoded NAbs were more potent compared with other NAbs and also displayed lower SHMs [33,34,35,36]. The IMGT/V analysis indicates that the IGHV of D6 is 7.37% somatically mutated at the nucleotide sequence level, which results in ten amino acid changes from the germline sequence, whereas the IGKV of D6 is 3.94% somatically mutated, resulting in four amino acid changes from its germline sequence. And the lengths of the HCDR3 and LCDR3 loops are 11 aa and 9 aa, respectively (Table 3, Figure 3). The protein size of D6’s heavy and light chains was determined by denatured SDS-PAGE (Figure 4F).

### 3.4. Binding Activity of D6 with RBD

To determine the binding activity of D6 to RBD, we performed ELISA experiments. We found D6 could specifically bind to prototype RBD, Delta RBD and BA.2 RBD, with the end-titers of 0.1280 ng/mL, 0.0256 ng/mL and 3.2000 ng/mL, respectively. VRC01, as a negative control, did not bind to prototype RBD, Delta RBD and BA.2 RBD (Figure 4A–C). These data indicate that D6 is an RBD-targeting mAb. 

### 3.5. The Binding Kinetics of D6 to RBD

An Octet RED96 system was used to test the binding kinetics of D6 with prototype RBD and BA.1 RBD. The KD values of D6 binding with prototype RBD and BA.1 RBD were both less than 1.0 × 10^−12^, and the Koff values of D6 binding with prototype RBD and BA.1 RBD were both less than 1.0 × 10^−7^. The Kon values of D6 binding with prototype RBD and BA.1 RBD were 2.95 × 10^5^ ± 2.31 × 10^3^ (1/Ms) and 1.55 × 10^5^ ± 9.07 × 10^2^ (1/Ms), respectively (Figure 4D,E). These data suggest that D6 could potently bind with RBD.

### 3.6. Neutralizing Activity of D6 against SARS-CoV-2 Pseudovirus Variants

**Figure 5:**
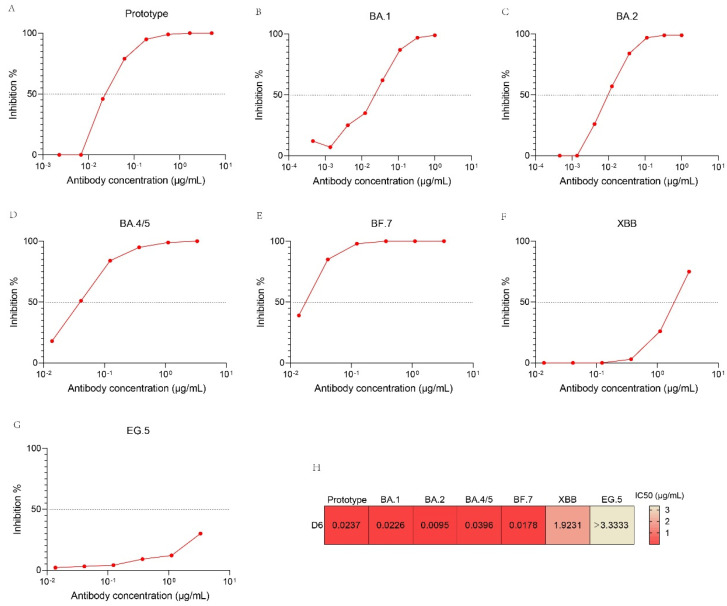
Neutralization activities of D6 against SARS−CoV−2 variants pseudoviruses, including the prototype (**A**), BA.1 (**B**), BA.2 (**C**), BA.4/5 (**D**), BF.7 (**E**), XBB (**F**) and EG.5 (**G**). Heat map of neutralization activities (IC50 values) of D6 against SARS-CoV-2 variant pseudoviruses (**H**).

To determine the neutralizing activity of D6 against SARS-CoV-2 variants, we performed pseudovirus neutralization assays against recently circulating virus variants, e.g., BA.1, BA.2. BA.4/5, BF.7, XBB and EG.5 (Figure 5). D6 could potently neutralize BA.2, with the lowest IC50 value of 0.0095 µg/mL (Figure 5C,H). D6 also could neutralize prototype, BA.1, BA.4/5, BF.7 and XBB, with IC50 values of 0.0237 µg/mL, 0.0226 µg/mL, 0.0396 µg/mL, 0.0178 µg/mL and 1.923 µg/mL, respectively (Figure 5A,B,D–F,H), but it failed to neutralize EG.5, with an IC50 value of more than 3.3333 μg/mL (Figure 5G,H).

### 3.7. Neutralization Activity of D6 against Authentic SARS-CoV-2 Variants

**Figure 6:**
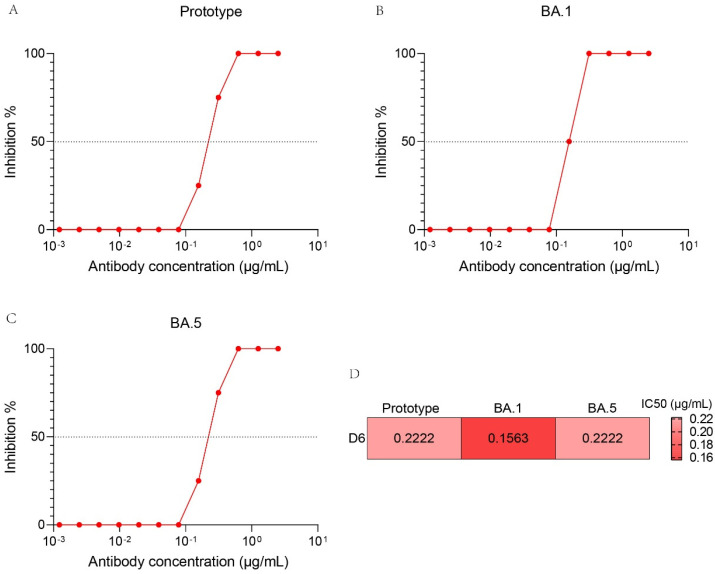
Neutralization activities of D6 against authentic SARS−CoV−2 variants, including the prototype (**A**), BA.1 (**B**), BA.5 (**C**). Heat map of neutralization activities (IC50 values) of D6 against authentic SARS-CoV-2 variants (**D**).

To further determine the neutralizing activity of D6 against SARS-CoV-2 variants, we also performed authentic SRAS-CoV-2 virus neutralization assays against the prototype, BA.1 and BA.5 strains, which were evaluated by a CPE-based method on Vero cells. D6 exhibited moderate neutralizing activity for the prototype, BA.1 and BA.5, with IC50 values of 0.2222 μg/mL, 0.1563 μg/mL and 0.2222 μg/mL, respectively (Figure 6A–C).

### 3.8. Structural Basis for Neutralization

To gain insight into the structural basis for the neutralizing mechanism of D6, the protein structure models of the D6 variable regions of heavy and light chains were built on the I-TASSER website excluding CC12.1 structures from I-TASSER template library, and the CC12.1-RBD complex crystal structure (PDB: 6XC2) was downloaded from the Protein Data Bank. 

MAb CC12.1 was selected as a control antibody due to several reasons. Firstly, the amino acid sequences of CC12.1 have high consistency with D6. There are only nine aa and 6 aa differences in the heavy and light chain (except the CDR3 loop) between D6 and CC12.1, respectively (Figure 3). CC12.1 and D6 are members of a shared genetic clonotype coming from the same germline genes, including IGHV3-53*01, IGHJ6*02, IGKV1-9*01 and IGKJ3*01 (Table 3). And they both have the NY motif and SGGS motif in the HCDR1 and HCDR2 loop, respectively. Therefore, D6 and CC12.1 are likely to have similar structures. Secondly, CC12.1 is a potent SARS-CoV-2 RBD-targeting NAb without cross-neutralizing activity. CC12.1 could potently neutralize the prototype pseudovirus, with an IC50 value of 0.019 µg/mL, but failed to neutralize Omicron subvariants, such as BA.1, BA.2 and BA.3, with IC50 values of more than 100 µg/mL [26,37]. Thirdly, the cry-electron microscopy structure of CC12.1-RBD has been determined [33]. If we found structure differences between CC12.1 and D6 interaction with RBD, we could identify the reason D6 cross-neutralizing activity against Omicron subvariants.

**Figure 7:**
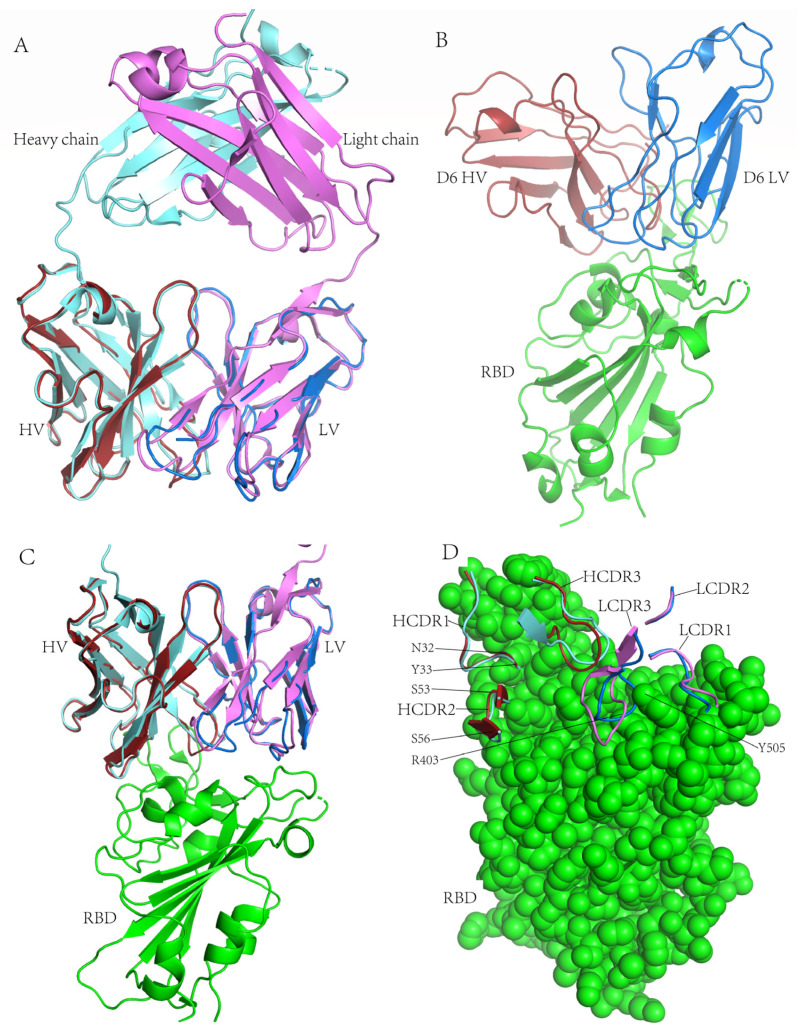
The structure of D6 and CC12.1 binding to SARS-CoV-2 RBD. (**A**) Diagram comparing the structures of D6 Fab and CC12.1 Fab. D6 Fab has a high structural similarity to CC12.1 Fab. The D6 heavy chain is shown in dark red, the light chain in blue. The CC12.1 heavy chain is shown in cyan, the light chain in magentas. (**B**) The structure of D6 Fab-RBD complex. The D6 heavy chain is shown in dark red, the light chain in blue. RBD is coloured in green. (**C**) The structure of D6 Fab is superimposed onto the CC12.1-RBD complex (PDB ID: 6XC2) by PyMOL2.4.1 software, with the CC12.1 structure as the reference. The RBD is coloured in green. (**D**) The CDRs of D6 and CC12.1 are displayed when bound to RBD. RBD is shown in green spheres. The HCDRs and LCDRs of D6 are shown in dark red and blue, respectively. The HCDRs and LCDRs of CC12.1 are shown in cyan and magenta, respectively.

The structure comparison showed that D6 Fab and CC12.1 Fab were highly consistent (Figure 7A). Then we superimposed D6 Fab onto the CC12.1-RBD complex, with CC12.1 Fab as the reference (Figure 7C). D6 bound to RBD with a similar angle of approach to CC12.1, hinting that D6 belongs to the RBD-1 community and only binds to RBDs in the ‘up’ conformation. In the D6-RBD complex, the HCDR1, HCDR2, HCDR3, LCDR1 and LCDR3 of D6 were all involved in interaction with the RBD external subdomain. The main differences between D6 and CC12.1 in combination with RBD were seen for HCDR3 and LCDR3. The D6 HCDR3 loop twisted about 45° relative to CC12.1 HCDR3 and touched the RBD deeper. The LCDR3 loop of the D6 also twisted relative to CC12.1 LCDR3, and went deep into the gap formed by R403 and Y505 of RBD (Figure 7B,D). The sufficient contact of D6 HCDR3 and LCDR3 with RBD may be the crucial reason for its cross-neutralizing activity.

## 4. Discussion

Omicron and its sublineages underwent a significant shift compared with the wild strain [38], often displaying enhanced transmissibility, and reduced vaccine effectiveness and antibody neutralizing activity, as well as increased likelihood of reinfection [39]. Especially, the occurrence of recombination when different variants are co-infected, such as XBB, which is a recombinant strain of BA.2.10.1 and BA.2.75 [40], allows the virus to acquire a broader phenotype and have more opportunities to escape immune stress [41]. Therefore, new and effective neutralizing antibodies against circulating Omicron variants and future pandemic variants of SARS-CoV-2 are urgently needed.

The heavy chain variable region of NAb D6 is encoded by the germline gene IGHV3-53, which is the most frequently used IGHV gene for RBD-targeting and ACE2 blocking human NAb [33,42]. Among our 57 mAbs, 10 of the VHs come from the IGHV3-53*01 germline, and 8 of these 10 mAbs could potently bind to prototype RBD. These data reconfirm that the IGHV3-53 germline is a very common germline among SARS-CoV-2 RBD-targeting mAbs. 

D6 is highly similar to the previously reported canonical antibody CC12.1 (Figure 7A). D6 and CC12.1 not only have the same variable region germline of heavy chain and light chain (IGHV3-53*01 and IGKV1-9*01), but also have same joining regions with germline of heavy chain and light chain (IGHJ6*02 and IGKJ3*01) (Table 3). And they both have short lengths of the HCDR3 loop (both 11 amino acids). It has been reported that the short HCDR3 (<15 amino acids) is to adapt the relatively flat IGHV3-53 antibody epitope, which presents a small pocket that only a short CDR3 loop could insert into [43]. The differences between D6 and CC12.1 are that they have different SHMs and have different IGHD germline genes. The VH and VK sequences of CC12.1 both acquired four amino acid mutations compared with their respective original germline sequences during affinity maturation (Figure 3), with SHM values of 1.05% and 1.08%, respectively, while the SHM of D6 VH and VK are 7.37% and 3.94%, respectively (Table 3). In addition, D6 has the NY motif at residues 32 and 33 in HCDR1 and has the SGGS motif at residues 53 to 56 in HCDR2, like CC12.1, which may strengthen the binding of D6 with RBD. 

In the BLI experiment, the KD value of D6 to prototype RBD was less than 1.0 × 10^−3^ nM, lower than CC12.1 to prototype RBD, with a value of 17 nM [43], which suggests that D6 had a higher affinity to RBD than CC12.1. The pseudovirus neutralization experiments showed that D6 exhibited potent neutralizing activity against the prototype, BA.1, BA.2 and BA.4/5, with an IC50 value of less than 0.040 µg/mL. CC12.1 could neutralize prototype, with an IC50 value of 0.019 μg/mL, and failed to neutralize Omicron [26]. The antibody structure showed that the largest difference between D6 and CC12.1 lies in the HCDR3 and LCDR3 loops, which was caused by only a few amino acid mutations. For example, HCDR3 and LCDR3 in D6 both twist at certain angles compared with CC12.1, and LCDR3 in D6 has a deeper contact with RBD than CC12.1 LCDR3. These differences in structure may be an important reason for the cross-neutralizing activity of D6. And these mutations of D6 compared with CC12.1 may provide important information for the function modification of SARS-CoV-2 Nabs.

D6 exhibited potent neutralizing activity against BA.2, with an IC50 value of 0.0095 µg/mL, 202-fold higher than XBB. Compared with its progenitor BA.2, XBB carries nine additional mutations (G339H, R346T, L368I, V445P, G446S, N460K, F486S, F490S, R493Q) in its RBD [44]. And it has been reported that the R346 position is a critical mutation site that leads to increased immune evasion by neutralizing antibodies [18,45,46,47]. However, the residue 346 of RBD does not interact with D6 directly in the D6-RBD complex structure. Thus, whether the decrease in neutralizing activity of D6 is due to one mutation or to the synergistic effect of R346 with other mutations needs to be further verified by mutation experiments. In addition, the binding of D6 to BA.2 RBD was weaker than to prototype and Delta RBD in the ELISA experiments. However, the neutralizing potency of D6 against BA.2 was higher than for the prototype and other subvariants. These findings may indicate that the neutralizing activity of mAb is not always directly related to its binding activity, which needs further study.

As far as neutralization against BA.1, BA.2 and BA.4/5 is concerned, the neutralizing activity of D6 exceeds 90% of the 1640 RBD-targeting mAbs reported by Cao et al. [17]. And D6 is comparable to several potent mAbs selected by Ju et al. [48], such as P5-1C8, P5S-2B10, P5S-2B6 and P5-1H1, coming from an IGHV3-53 germline such as D6. However, the neutralizing activity of D6 against the emerging epidemic strains decreased greatly.

D6 was isolated from one of the earliest COVID-19 convalescents who was infected with the prototype SARS-CoV-2 and received inactivated COVID-19 vaccine of the prototype. However, D6 has cross-neutralizing activity against several Omicron sublineages. This may be for two reasons. One is the donor was vaccinated after infection when the immune system was boosted. The other is that the donor had spent a long time recovering before the blood was sampled, which may have provided NAbs more time to evolve.

In conclusion, we isolated a SARS-CoV-2 RBD-specific cross-neutralizing mAb D6. D6 could efficiently neutralize the prototype strain and several circulating Omicron subvariants such as BA.1, BA.2, BA.4/5, BF.7 and XBB. The sorting and analysis of mAb D6 will provide important information for the development of anti-COVID-19 reagents.

## Figures and Tables

**Table 1:** The profile of the donor YYQ.

Donor	Age	Gender	Infection Time	Clinical Symptom	Hospitalization Length (Days)	Vaccine Type after Infection	Sampling Date
YYQ	35	Male	22 January 2020	Mild	10	Inactivated vaccine	16 December 2021

**Table 2:** The neutralizing activity of YYQ plasma (ID50).

Pseudovirus	Authentic Viruses			
Prototype	Prototype	Beta (B.1.351)	Gamma (P.1)	Kappa (B.1.167.1)
540	384	12	4	16

**Table 3:** Genetic analysis of Ab D6 and CC12.1 by the IMGT/V-Quest program. CC12.1 is a previously reported potent RBD-directed NAb whose gene sequences are very similar to those of D6.

	Heavy Chain					Light Chain			
	IGHV	IGHD	IGHJ	CDR3 (aa)	SHM (%)	IGKV	IGKJ	CDR3 (aa)	SHM (%)
D6	3-53*01	2-8*01	6*02	11	7.37	1-9*01	3*01	9	3.94
CC12.1	3-53*01	5-24*01	6*02	11	1.05	1-9*01	3*01	11	1.08

## Data Availability

The data presented in this study are available in this published article and its Supplementary Table S1.

## Supplementary Materials

The following supporting information can be downloaded at: https://www.mdpi.com/article/10.3390/pathogens13040272/s1; Table S1: The binding activity of 57 mAbs to Prototype and Omicron respectively (OD450 value).
