# Supplementary material for: Identification of an IGHV3-53-Encoded RBD-Targeting Cross-Neutralizing Antibody from an Early COVID-19 Convalescent
Source: Pathogens. 2024 Mar 23;13(4):272. doi: 10.3390/pathogens13040272 (PMC11054858; doi:10.3390/pathogens13040272)
Supplement: Supplementary file 1 [file pathogens-13-00272-s001.zip › pathogens-2869764-supplementary.pdf]

Table S1. The binding activity of 57 mAbs to Prototype and Omicron respectively (OD_{450} value).

MAbs	Prototype	Omicron
A1	1.2210	0.0180
A2	0.0045	0.0060
A3	3.0105	0.0235
A8	2.4265	0.0085
A10	0.1065	0.0080
A12	0.7420	0.0065
B4	0.0065	0.0060
B8	2.8225	0.0075
B10	2.9920	0.5200
B11	0.1980	0.0090
B12	3.1335	0.0175
C1	2.6550	0.0095
C3	3.0165	1.1530
C6	2.9040	0.5105
C8	0.4090	0.2435
C10	0.9925	0.0060
C11	3.0730	0.2135
C12	3.0330	0.5615
D6	3.0890	2.4625
D7	3.0585	0.0165
D9	0.9755	0.0165
E2	3.1790	0.0070
E4	3.3065	0.0700
E5	2.7205	0.1890
E7	0.0050	0.0045
E9	2.4015	0.0315
E10	2.3810	0.0065
E12	2.9965	0.0060
F1	0.0135	0.0070
F5	0.6975	0.6970
F8	3.0090	0.6880
F9	2.8630	0.0145
F11	1.1195	0.0145
G2	2.9730	0.9865
G3	3.0910	2.2395
G5	0.2855	0.0565
G6	2.8550	0.0070
G8	3.1020	0.0120
G11	3.0410	0.0070
G12	3.3185	2.1955
H2	2.6520	0.0115
H4	2.9215	0.6680
H5	2.8980	0.0105
H6	2.6255	0.0070
H8	2.7840	0.0430
H10	2.7650	0.0065
H11	0.0390	0.0075
H12	0.0245	0.0095
A4-2	2.2115	0.0060
A5-2	0.0055	0.0050
A6-2	0.0935	0.0070
A7-2	2.3835	0.1775

A12-2	2.1760	0.0650
B7-2	2.7220	0.0165
B8-2	2.4860	0.1450
B9-2	2.9330	0.0150
B11-2	2.7135	0.0100
Negative control	0.0075	0.0060

- ✧ MAb was expressed in 24-well plate in duplicate wells.
- ✧ The OD_{450} value was the average value of two independent ELISA experiments.
- ✧ Nine mAbs in light orange background were tested neutralizing activity against pseudovirus of prototype stain.
